# Machine Learning Based on Multi-Parametric MRI to Predict Risk of Breast Cancer

**DOI:** 10.3389/fonc.2021.570747

**Published:** 2021-02-26

**Authors:** Weijing Tao, Mengjie Lu, Xiaoyu Zhou, Stefania Montemezzi, Genji Bai, Yangming Yue, Xiuli Li, Lun Zhao, Changsheng Zhou, Guangming Lu

**Affiliations:** ^1^ Department of Medical Imaging, Jinling Hospital, Medical School of Nanjing University, Nanjing, China; ^2^ Department of Nuclear Medicine, The Affiliated Huai’an No. 1 People’s Hospital of Nanjing Medical University, Huai’an, China; ^3^ Faculty of Mechanical Electronic and Information Engineering, Jiangsu Vocational College of Finance and Economics, Huai’an, China; ^4^ Radiology Unit, Department of Pathology and Diagnostics, Azienda Ospedaliera Universitaria Integrata-Verona, Verona, Italy; ^5^ Department of Radiology, The Affiliated Huai’an No. 1 People’s Hospital of Nanjing Medical University, Huai’an, China; ^6^ Deepwise AI Laboratory, Deepwise Inc., Beijing, China

**Keywords:** breast cancer, multi-parametric MRI, machine learning, risk prediction, nomogram

## Abstract

**Purpose:**

Machine learning (ML) can extract high-throughput features of images to predict disease. This study aimed to develop nomogram of multi-parametric MRI (mpMRI) ML model to predict the risk of breast cancer.

**Methods:**

The mpMRI included non-enhanced and enhanced T1-weighted imaging (T1WI), T2-weighted imaging (T2WI), apparent diffusion coefficient (ADC), *K* ^trans^, *K*_ep_, *V*_e_, and *V*_p_. Regions of interest were annotated in an enhanced T1WI map and mapped to other maps in every slice. 1,132 features and top-10 principal components were extracted from every parameter map. Single-parametric and multi-parametric ML models were constructed *via* 10 rounds of five-fold cross-validation. The model with the highest area under the curve (AUC) was considered as the optimal model and validated by calibration curve and decision curve. Nomogram was built with the optimal ML model and patients’ characteristics.

**Results:**

This study involved 144 malignant lesions and 66 benign lesions. The average age of patients with benign and malignant lesions was 42.5 years old and 50.8 years old, respectively, which were statistically different. The sixth and fourth principal components of *K* ^trans^ had more importance than others. The AUCs of *K* ^trans^, *K*_ep_, *V*_e_ and *V*_p_, non-enhanced T1WI, enhanced T1WI, T2WI, and ADC models were 0.86, 0.81, 0.81, 0.83, 0.79, 0.81, 0.84, and 0.83 respectively. The model with an AUC of 0.90 was considered as the optimal model which was validated by calibration curve and decision curve. Nomogram for the prediction of breast cancer was built with the optimal ML models and patient age.

**Conclusion:**

Nomogram could improve the ability of breast cancer prediction preoperatively.

## Introduction

Breast cancer is the most frequently diagnosed malignancy among women worldwide and accounts for 30% of all new cancer cases in 2018 (1). It is estimated that one in eight women will be diagnosed with breast cancer in their lifetime and that one patient will be diagnosed with breast cancer every 10 min (2). Therefore, the diagnosis of breast cancer is crucial for every patient with breast lesions.

Dynamic contrast-enhanced MRI (DCE-MRI) is a powerful tool for detecting breast cancer. It has the highest sensitivity of all methods for the examination of breast lesions (3). However, because of an overlap of the morphologic and kinetic features of benign and malignant lesions, the limited specificity of DCE-MRI could lead to the misdiagnosis of malignant lesions, overtreatment of benign lesions, and unnecessary breast biopsies and surgery (4, 5). Therefore, it is necessary to combine multi-parametric MRI (mpMRI), which includes conventional sequences such as pre-enhanced and enhanced T1-weighted imaging (T1WI) and T2-weighted imaging (T2WI), and also function sequences such as diffusion weighted imaging (DWI) and pharmacokinetic DCE (Pk-DCE) to improve diagnosing efficiency in breast cancer.

Nevertheless, not every parameter in mpMRI will increase the diagnostic specificity of breast cancer. Given the huge amount of information offered by mpMRI, it is challenging to select the most effective method for the diagnosis of breast cancer in humans (6). Machine learning (ML), which is a branch of artificial intelligence, can extract high-throughput features of lesions that are imperceptible to human eyes, learn the characteristics of lesions, and make a diagnosis based on them using computer-aided technology (7–13). Although, ML on mpMRI is widely under investigation for the diagnosis of breast lesions (8, 9, 14–23), there are few studies that focus on nomogram of the optimal mpMRI model to diagnose breast cancer. In this study, our aim is to provide an effective nomogram of the optimal ML model based on mpMRI for the risk prediction of breast cancer preoperatively.

## Materials and Methods

### Patient Inclusion Criteria

This study was approved by our institution and informed consent was obtained from all study participants. The patients were selected based on the inclusion criteria as follows: 1) all patients underwent an MRI examination before treatment or biopsy; 2) breast lesions were identified as primary malignant and benign lesions *via* pathology; 3) the mpMRI for every patient enrolled must include consistent sequences, pre-enhanced and enhanced T1WI, T2WI, DWI, and Pk-DCE. The exclusion criteria were the following: 1) the patient was undergoing biopsy or surgery at an external institution and the pathological results were not available; 2) the Pk-DCE sequence could not be processed to generate pharmacokinetic parameter maps; 3) DWI sequence could not generate clear apparent diffusion coefficient (ADC) map; and 4) lesions on the image could not be identified so that they could not be annotated.

### Pathological Examination

All patients underwent surgical resection, and the removed specimens were fixed in formalin and embedded with paraffin. The specimens were stained with hematoxylin and eosin to determine the histological type. According to the Scarff, Bloom, and Richardson histologic grade, invasive ductal carcinoma of breast cancer was classified as Grade I, Grade II, or Grade III (24). All pathological diagnoses were obtained by two experienced pathologists.

### MRI Protocol

MRI was performed with the patient lying in the prone position, with 4-channel bilateral breast coils covering both breasts on a 3.0 T MRI scanner (Verio, Siemens Healthcare, Erlangen, Germany). The parameters of the sequences were as follows: (1) T2WI with a repetition time/echo time (TR/TE) of 4,300/61 ms, 34 slices, a field of view (FOV) of 340–400 mm, a slice thickness of 4 mm, and an acquisition time of 2 min 45 s; (2) DWI with a TR/TE of 7,100/95 ms, b = 50 and 800 s/mm^2^, 24 slices, an FOV of 320–380 mm, a slice thickness of 4 mm, and an acquisition time of 3 min 12 s; (3) axial, vibe fat-suppressed T1WI with a TR/TE of 3.61/0.96 ms; flip angles of 3°, 6°, 9°, 12°, and 15° successively, 30 slices, an FOV of 380–420 mm, a slice thickness of 4 mm, and an acquisition time of 8 s per scan. The next similar sequence with a flip angle of 12° was performed for 40 scans continuously, and after the second period, MRI contrast agent Omniscan (GE Healthcare Co., Ltd.) with 0.2 ml/kg was injected into an antecubital vein *via* a catheter at rate of 2 ml/s using a power injector (Medrad, Warrendale, PA), followed by a 20 ml saline flush.

### Pk-DCE Data Processing

Pk-DCE datasets were processed using Omni-Kinetics (O.K.) software (GE Healthcare). First, the acquired Pk-DCE images were processed by a Markov-random-field based three-dimensional non-rigid registration algorithm to correct for the patient motion that occurs between the phases of dynamic data due to respiration and other involuntary movements. Second, the individual arterial input function was obtained from a region of interest (ROI) in the thoracic aorta. Third, using an extended Tofts model, *K* ^trans^, *K*_ep_, *V*_e_, and *V*_p_ maps were obtained for each case (25).

### ROI Segmentation

All the maps were preprocessed to ensure that their matrices were consistent. ROIs were segmented manually in the enhanced-T1WI map over the entire lesion to avoid the partial volume effect and exclude necrosis, cystic areas, and vessels using ITK-SNAP software (version 3.0; www.itksnap.org) separately by two radiologists who had more than 10 years of experience in breast MRI analysis. If the boundary of tumor area was blurred or the results were inconsistent, two radiologists negotiate each other and reached a consensus. The maximum diameter of the lesion was measured in the enhanced-T1WI map to compare the lesion sizes. The ROIs in the enhanced-T1 weighted map were firstly resampled and transformed to physical space which was shared by all maps. Then the ROIs in physical space were transformed back to voxel space of other maps. The radiologist would tweak and exam other maps (*i.e.*, the non-enhanced T1WI, T2WI, ADC, *K* ^trans^, *K*_ep_, *V*_e_, and *V*_p_) to make sure ROIs’ right place. This step was completed using SimpleITK (Version 1.2.0, http://www.simpleitk.org).

### ML and Statistical Analysis

#### Radiomics Features Extraction

Radiomics features extraction was performed using Pyradiomics (26) (Version 2.1.0). With this package, a total of 1132 features of each lesion were extracted from all eight MRI parameter maps, including first-order, shape, and texture features.

### Principal Component Analysis of Features Extracted by Random Forest Model

The top-10 principal components from each MRI parameter were extracted construct a random forest model with the breast lesion labels of benign and malignant. The top 22 out of 80 principal components in coefficient ranking were chosen to analyze by the method of Random forest models with the breast lesion labels of benign and malignant. All of the above steps were performed in Python 3.6.

### Feature Selection

To avoid overfitting, feature selection was implemented. In this stage, different statistical methods were applied to features to calculate the scores. And features were selected or removed according to their rank. F-test, Pearson Correlation Coefficient, Mutual information, L1 based method, Tree based method and Recursive Feature Elimination were used in our study. Feature selection was implemented using the Deepwise Research Platform (https://research.deepwise.com/).

### Single-Parameter ML Models

Twelve classifiers (Logistic Regression, SVM, Linear SVC, Decision Tree, Random Forest, Ada Boost, Bernoulli NB, Gaussian NB, K Nearest Neighbors, Linear Discriminant Analysis, SGD and Multilayer Perceptron) were used to construct discriminative models for each MRI parameter with features selected. Models were trained and validated for 5-fold cross-validation with 10 rounds on all the lesions using the Deepwise Research Platform. The final performance of each single-parameter model was average of the all models from all ten rounds. The area under the receiver operating characteristic (ROC) curve (AUC) values of the model was calculated using ground truth label and score. The AUC, accuracy, specificity, and sensitivity of the eight single-parameter models were evaluated to determine their diagnostic efficiency for breast cancer.

### Multi-Parametric ML Models

**Figure 1:**
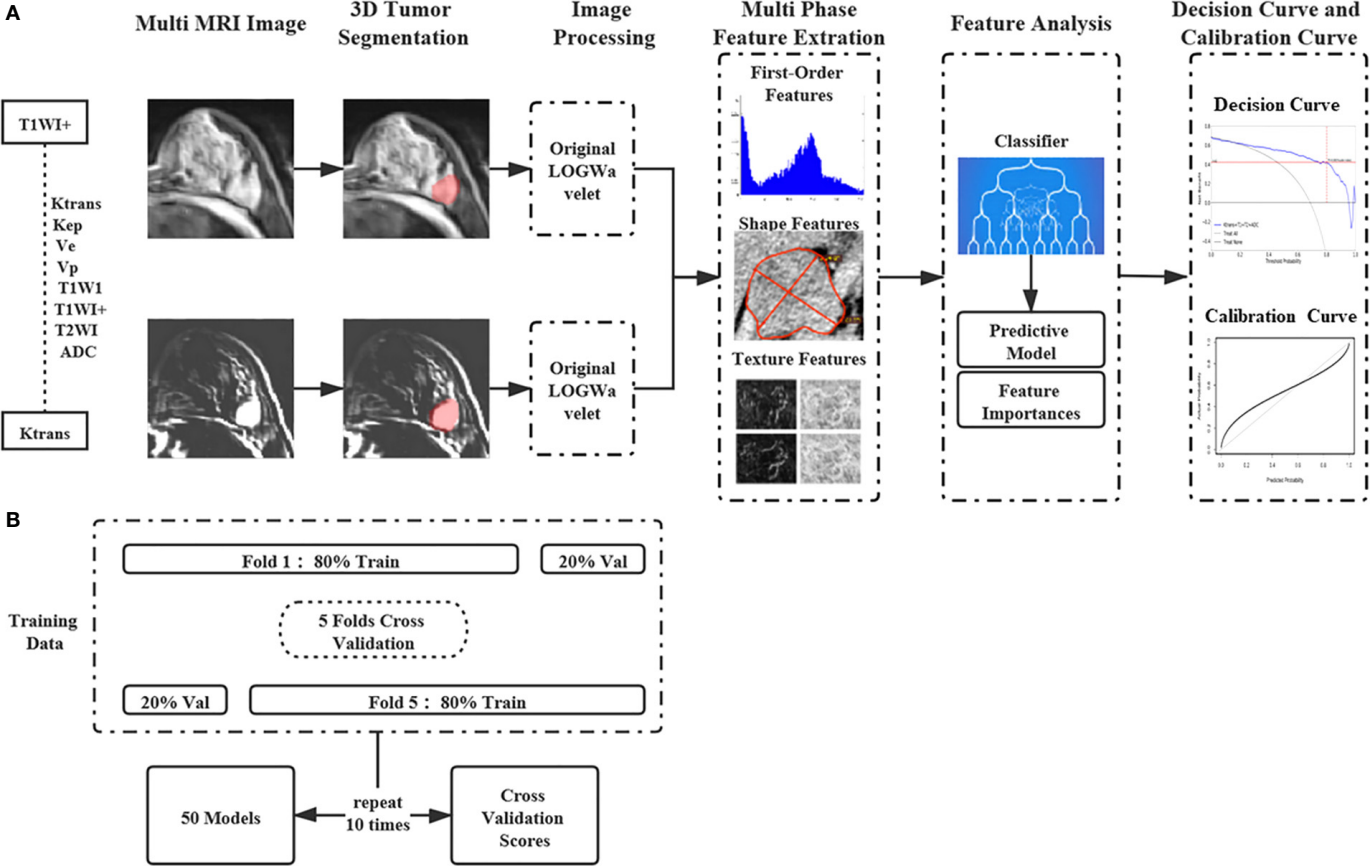
The machine learning workflow with the features of multi-parametric MRI images. **(A)** The features of the single and Multi-parametric MRI parametric images are extracted and selected to construct machine learning models. **(B)** Machine Learning Models were trained and validated for 5-fold cross-validation with 10 rounds on all the lesions.

The appropriate classifiers were chosen to construct a multi-parametric model based on the results of the single-parametric ML models. There were 247 potential combinations of eight MRI parameters. In addition to classifier selection, models were also trained and validated for 5-fold cross-validation with 10 rounds on all the lesions using the Deepwise Research Platform. The average of the 10 verification scores from all 10 rounds was used as the final verification score of every multi-parametric ML model. Same metrics were used to evaluate the diagnostic efficiency as single-parameter ML methods. For the multi-parametric ML models, the one with highest diagnostic efficiency was determined to be the optimal ML model. The overall ML scheme was described in **Figure 1**, which showed how the features of the MRI parametric images are handled.

### Validation of the Optimal ML Model

The performance of the validation was tested in the validation cohort. The value of the optimal ML model as a predictor of benign and malignant lesions was evaluated with the index of consistency in calibration curve. The net benefit was calculated by subtracting the proportion of all patients who were false positive from the proportion who were true positive in the decision curve.

The calibration curve was created using RMS package in RStudio 1.1., while the decision curve was plotted in python 3.6 with matplotlib package.

### Breast Cancer Risk Model Built by Nomogram

The clinicopathologic characteristic of breast lesions were statistically analyzed using the software of GraphPad Prism 6. A parametric test (unpaired t‐test) was applied when normality assumptions and homogeneity of variance were satisfied. Otherwise, the equivalent non‐parametric test (Mann–Whitney test) was used. P < 0.05 indicated statistical significance. The clinicopathologic characteristics with statistical difference between benign and malignant lesions and the optimal ML model were used to construct breast cancer risk model. Nomogram was created to construct the risk model to assess the risk of breast cancer using the RMS package in RStudio 1.1.

## Results

### Enrolled Patients

**Figure 2:**
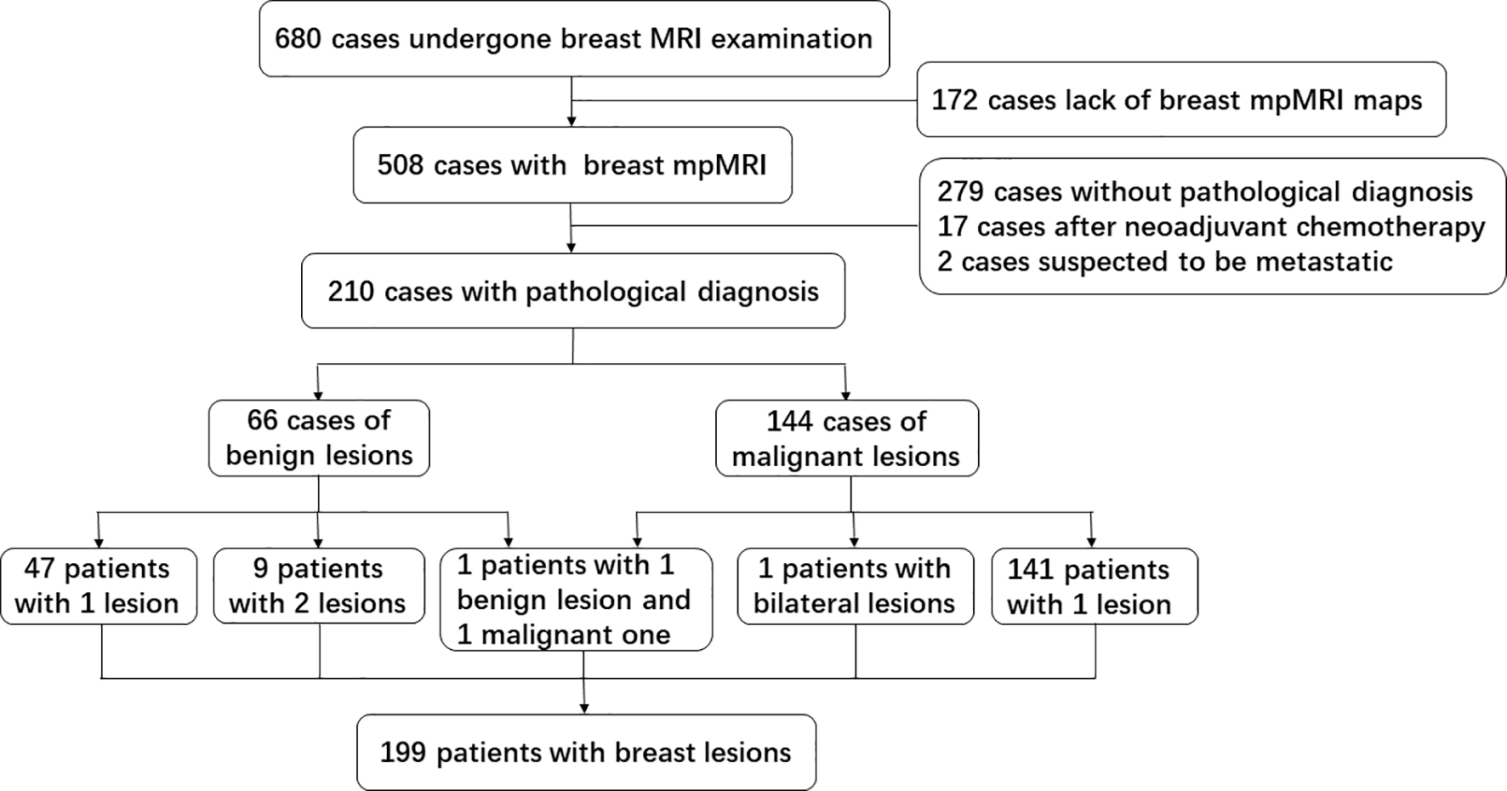
Flow diagram of inclusion and exclusion about the cohort data in this study.

As shown in **Figure 2**, from October 2016 to June 2018, 210 breast lesions (144 malignant lesions and 66 benign lesions) from 199 female patients met the above criteria and were enrolled in the study. There were nine patients with two benign lesions, one patient with bilateral malignant lesions, and one patient with 1 benign lesion and 1 malignant lesion. The clinicopathologic characteristics of patients, including age, lesion size, pathological type and histologic grade, were summarized in **Table 1**. The average age of patients with benign and malignant were 42.5 years old and 50.8 years old respectively, which had statistically significant difference. The size of the benign lesions was not significantly different from that of the malignant lesions.

**Table 1:** Clinicopathologic characteristics of the study cohort.

Classification	Age (mean ± SD), years	Lesion size, [media(Q_25_, Q_75_], cm	Histologic type/grade	
**Benign lesions** (n = 66)	42.5 ± 11.0	2.8[1.6, 4.4]	Fibroadenoma (n=21)	
			Adenosis (n=14)	
			Others (n=31)	
**Malignant lesions** (n=144)	50.8 ± 9.5	3.3[2.2, 4.8]	IDC (n=121)	I (n=8)
				II (n=86)
				III(n=27)
			Others (n=23)	
**P**	<0.001	0.081	/	

IDC, invasive ductal carcinoma.

### The Sixth Principal Component of K^trans^ Was the One With the Highest Importance

**Figure 3:**
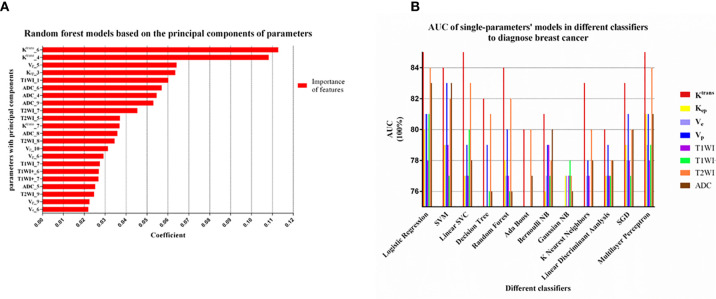
**(A)** The principal component analysis of each parameter by Random forest model. The top-10 principal components of every parameter were extracted. The top 22 out of 80 principal components in coefficient ranking were chosen to construct a model by the method of Random forest model. The sixth and fourth principal component of *K* ^trans^ were obviously more important than other principal component of parameters. **(B)** Machine learning models with 12 classifiers were constructed to select the appropriate classifier according to the AUC of the single parameter models. The models with Logistic Regression, SVM, Multilayer Perceptron had relatively high AUC values (>0.80) and this three classifiers were considered as appropriate classifiers in our study.

The top-10 principal components of every parameter were extracted from 1,132 features to construct a random-forest model, which had AUC of 0.84 and accuracy of 0.81. In this model, the sixth and fourth principal component of *K* ^trans^ were about twice as important as the other principal components (**Figure 3A**).

### Single-Parameter ML Model

#### The *K* ^trans^ ML Model Had the Best Discriminative Performance in the Single-Parametric ML Models

The models of *K* ^trans^, *K*_ep_, *V*_e_, *V*_p_, non-enhanced T1WI, enhanced T1WI, T2WI, and ADC maps had AUC values of 0.86, 0.81, 0.81, 0.83, 0.79, 0.81, 0.84, and 0.83 for the validation cohort, respectively. The sensitivity, specificity, and accuracy of each parameter model was listed in **Table 2**. The *K* ^trans^ ML model was the optimal single-parametric model with the best discriminative performance.

**Table 2:** The AUC, sensitivity, specificity, and accuracy of single-parameter models and the joint optimal model.

Parameters	Rad_Score[media(Q_25_, Q_75_]	AUC	Sensitivity	Specificity	Accuracy
**K^trans^**	0.84(0.46,0.93)	0.86	88%	74%	83%
**K_ep_**	0.78(0.48,0.90)	0.81	80%	67%	76%
**V_e_**	0.69(0.35,0.84)	0.81	76%	77%	77%
**V_p_**	0.96(0.18,1.24)	0.83	84%	74%	80%
**T1WI**	0.83(0.25,1.08)	0.79	78%	68%	77%
**T1WI+**	0.70(0.33,0.88)	0.81	80%	70%	76%
**T2WI**	0.66(0.34,0.84)	0.84	67%	88%	76%
**ADC**	0.80(0.46,0.92)	0.83	84%	74%	77%
**K^trans^_T1WI_T2WI_ADC**	0.90(0.34,0.98)	0.90	81%	89%	85%

#### Classifiers Selection During Single-Parametric ML Models

In unbalanced data, AUC was more appropriate than accuracy, sensitivity and specificity to evaluate the performance of a model. We can see from **Figure 3B** that the models using the classifiers of Logistic Regression, SVM and Multilayer Perceptron had higher AUC values (>0.80), which were considered to be relatively good classifiers for constructing the breast cancer discriminative model in this study. These three feature classifiers were chosen to analyze the combined multiple parameters in the next section.

### The Optimal mpMRI ML Model

**Figure 4:**
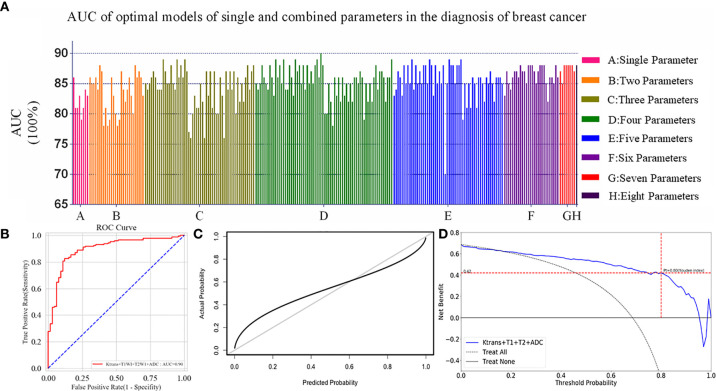
**(A)** All the models including single and multiply parameters were built with Logistic Regression, SVM and Multilayer Perceptron. One model combined with *K* ^trans^, non-enhanced T1WI, T2WI and ADC had an AUC of 0.90, and was highest in all experiments. **(B)** ROC curve of the optimal model was constructed by four parameters with the AUC value of 0.90 in the diagnosis of breast cancer. **(C)** Calibration curves of the optimal ML model for the diagnosis of breast cancer based on the mpMRI. The y-axis represented the actual probability. The x-axis represented the predicted probability. The diagonal gray line represented a perfect prediction by an ideal model. The black solid line represented the performance of the optimal ML model, of which a closer fit to the diagonal gray line represented a better prediction. **(D)** Decision curve analysis for the optimal ML model based on the mpMRI for the diagnosis of breast cancer. The y-axis measured the net benefit. The x-axis represented the threshold probability. The blue line represented the optimal ML model. The dotted line represented the assumption that all patients had breast cancer. Thin black line represented the assumption that no patients had breast cancer. The net benefit was calculated by subtracting the proportion of all patients who were false positive from the proportion who were true positive, weighting by the risk of malignant lesions compared with the lesions. According to the Youden index in the validation cohort, when the threshold probability (Pt) was 0.80, the net benefit was 0.42. When Pt was 0.17–0.95, the net benefit of the optimal model was better than the treat-all or treat-none strategies.

A total of 247 experiments with mpMRI ML models were performed using six feature selection methods and three classifiers. The AUC values of every single and multi-parametric models were shown in **Figure 4**. The model that combined of *K* ^trans^, non-enhanced T1WI, T2WI, and ADC, achieved the highest AUC value of 0.90 with multilayer perceptron as classifier in our study (**Figure 4B**).

**Figure 5:**
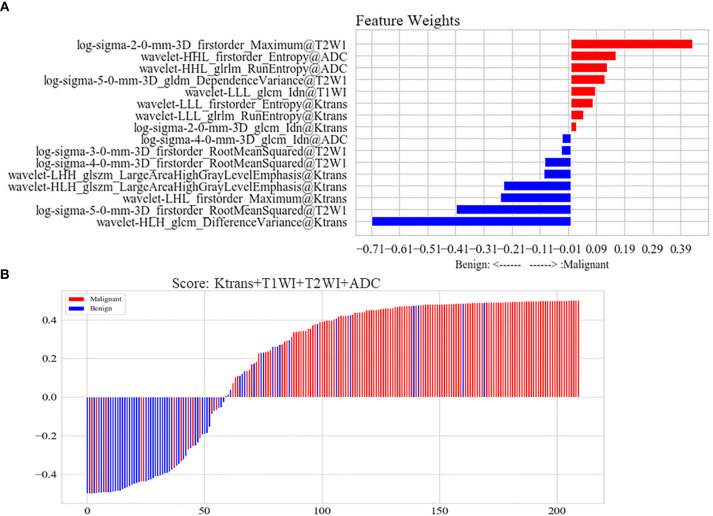
**(A)** Features of the optimal model constructed by *K* ^trans^, non-enhanced T1WI, T2WI, and ADC. The joint multi-parameter model was constructed by the top 16 features in the weight coefficient ranking of features. Red represented that the lesion with higher weight feature was possible of malignant lesion in the model. Blue represented that the lesion with higher weight feature was possible of benign lesion in the model. **(B)** Score of the optimal model constructed by *K* ^trans^, non-enhanced T1WI, T2WI and ADC for each lesion. Scores above zero meant malignant lesions in the model. Scores below zero meant benign lesions in model. Red represented malignant lesions in pathology, while blue represented benign lesions.

It was showed that the 16 high-weight features in the optimal model were chosen by a Pearson correlation analysis from the features of four MRI parameters, and *K* ^trans^ had seven features beyond the other three parameters in **Figure 5A**. Each lesion’s score predicted by the optimal ML model was displayed in **Figure 5B**. The sensitivity, specificity, and accuracy rates of the optimal ML model were 81%, 89%, and 85%, respectively (**Table 2**).

### Validation of the Optimal Model

The index of concordance in the optimal ML model was 0.90 for the calibration curve in **Figure 4C**. The decision curve in **Figure 4D** shows that the clinical usefulness of the optimal model on the validation cohort. A greater net benefit was obtained when the threshold probability (Pt) was between 0.17 and 0.95, comparing Treat-none or Treat-all. Besides, according to the Youden index in the validation cohort, when the Pt was 0.80, the corresponding result was 0.42 on the decision curve, which meant that the optimal model was able to detect 42 breast-cancer-positive patients per 100 patients without increasing false-positives.

### Nomograms of the Optimal ML Model

**Figure 6:**
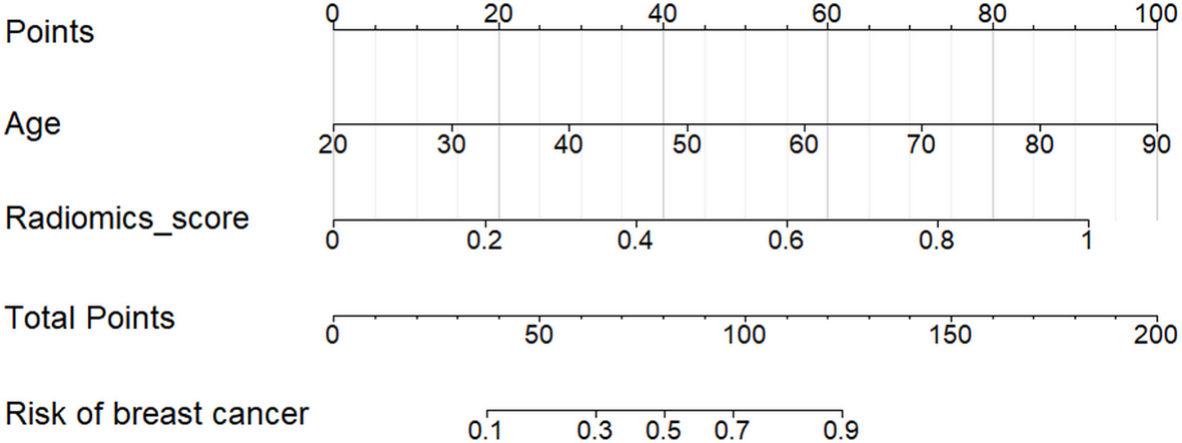
Nomogram for the risk prediction of breast cancer with patient age and the optimal ML model. The risk of breast cancer was positively correlated with the patient age and radiomic score of every lesion in the optimal ML model.

There was significant statistical difference in the patient age between benign and malignant lesions. The nomograms were built to predict the risk of malignant breast lesions by using the optimal ML model scores and patient age (**Figure 6**). In the nomogram, the older a person with breast disease and the higher radiomic score in the optimal ML model, the greater the risk of breast cancer.

## Discussion

In the present study, the principle components of *K* ^trans^ were more important than the principle components of other parameters, and the ML model of *K* ^trans^ map had the highest AUC, accuracy, and sensitivity of all single-parameter ML models. This indicated that Pk-DCE may be an important functional MRI with a high ability to identify breast cancer. The ML model combined of *K* ^trans^, non-enhanced T1WI, T2WI, and ADC had the highest AUC value, accuracy, and specificity as well as a higher sensitivity than the other single-parameter and multi-parameter models for the breast cancer diagnosis. This four-parameter ML model could improve the discrimination ability of breast lesions. The calibration curve and decision curve demonstrated that the optimal ML model has good clinical value. In addition, patient age was significantly different for benign and malignant lesions. We created nomograms by combining the optimal ML model and clinical data, which could be helpful in the prediction of breast cancer risk.

This study was the first ML research on breast mpMRIs with conventional and functional sequences. First, we noted that ML was superior to the previous diagnostic analysis because it can recognize texture features that hardly be found by human eyes. Furthermore, the functional MRI, especially Pk-DCE, was suitable for identifying breast cancer. Additionally, breast cancer can be diagnosed not only by single parameter but also by combinations of multiply parameter. However, we found that the optimal ML model was not a single-parametric model nor a model that combined all the parameters, but instead was a model that combines four parameters, *K* ^trans^, non-enhanced T1WI, T2WI, and ADC. The optimal ML model, with AUC 0.90, specificity 89% and accuracy 85%, was a good tool for preoperatively breast cancer diagnosing. The calibration curve and the decision curve analysis revealed that the optimal ML model was clinically useful. Nowadays, pathological diagnosis after surgery or biopsy was predominately used as the gold standard. However, sampling error was inevitable and invasive procedures may have adverse effects on patients (27).

The nomograms built by the optimal ML model could be beneficial for preoperatively predicting the risk of breast cancer in patients, enabling them to avoid unnecessary interventions for benign lesions.

The nomograms revealed that the optimal ML model was an important factor for predicting the risk of malignant breast lesions. In addition to the optimal ML model, the patient age played important roles in the prediction of breast cancer risk. The median age at breast cancer diagnosis was 61 years (28). Moreover, 81% of breast cancers were diagnosed among females 50 years old and older (29). In our study, the average age of patients with breast cancer was 50.8 years old. The age distribution in the literature was somewhat different to that of our study, which may be caused by the different samples.

There have been a few studies on breast mpMRI that applied conventional analytical methods (3, 25, 30–33). Truhn et al. used radiomics and deep learning to analyze breast mpMRI but the breast mpMRIs in their study only included T2WI and contrast-enhanced subtracted or non-subtracted images (23). In our study, we diagnosed breast lesions not only with conventional sequences, non-enhanced and enhanced T1WI as well as T2WI, but also with the functional sequences, DWI and Pk-DCE.

For the lesion segmentation of mpMRI images, the consistency of ROI annotation was a prerequisite to ensuring the accuracy of feature extraction (23). To improve the accuracy of lesion segmentation, we marked lesion ROIs on the enhanced T1WI image. We then matched this image and the lesion ROIs with the other multi-parametric images and the corresponding target areas using the software ITK-SNAP, whose powerful image processing facilitated the development of multiple MRI parameter-based ML methods.

This study had some limitations. First, the inherent limitation of ML was that the complex decision-making process of the model is difficult to understand, which resulted in ML often being called a “black box.” Additionally, the number of cases was relatively small and the appropriate external validation data were difficult to obtain. It was impossible to do a ML study based on histologic grade. In the next step, we plan to expand the sample for further study of ML methods in breast mpMRIs.

## Conclusion

The ML based on mpMRI with functional sequences in the current study could extracted more image features of breast lesions that naked eyes could detect and had certain advantages in the diagnosis of breast cancer over the traditional analysis method. The nomograms with the optimal ML model based on mpMRI will clearly improve the efficiency of breast cancer prediction.

## Data Availability Statement

The raw data supporting the conclusions of this article will be made available by the authors, without undue reservation.

## Ethics Statement

The studies involving human participants were reviewed and approved by the Affiliated Huai’an No. 1 People’s Hospital of Nanjing Medical University. The patients/participants provided their written informed consent to participate in this study. Written informed consent was obtained from the individual(s) for the publication of any potentially identifiable images or data included in this article.

## Author Contributions

Conceptualization: GL. Data curation: WT, XZ. Formal analysis: ML, YY. Methodology: WT, LZ. Resources: WT, GB. Supervision: XL, CZ. Visualization: LZ, YY. Writing—original draft: WT. Writing—review and editing: GL, SM. All authors contributed to the article and approved the submitted version.

## Funding

This study was funded by the grant from the Jiangsu Postdoctoral Foundation (grant no.2019K084 to WT) the Foundation of Nanjing Medicine University (grant no. NMUB2019351 to WT), Huai’an Science and Technology Project (grant no. HAB202017 to WT) and the Jiangsu Provincial Natural Science Foundation (grant no. 19KJB520025 to XZ).

## Conflict of Interest

YY, XL, and LZ were employed by the company of Deepwise.

The remaining authors declare that the research was conducted in the absence of any commercial or financial relationships that could be construed as a potential conflict of interest.

## Abbreviations

ML, machine learning; mpMRI, multi-parametric magnetic resonance imaging; PK-DCE MRI, pharmacokinetic dynamic contrast-enhanced magnetic resonance imaging; DWI, diffusion weighted imaging; ADC, apparent diffusion coefficient; T1WI, T1-weighted imaging; T2WI, T2-weighted imaging; PCA, Principal component analysis; ROC curve, receiver operating characteristic curve; AUC, area under curve; ROI, region of interest.
