# Supplementary material for: Machine Learning Based on Multi-Parametric MRI to Predict Risk of Breast Cancer
Source: Front Oncol. 2021 Feb 26;11:570747. doi: 10.3389/fonc.2021.570747 (PMC7952867; doi:10.3389/fonc.2021.570747)
Supplement: Supplementary file 1 [file Table_1.doc]

|  | Feat of features | score |
| --- | --- | --- |
| 1 | original_glszm_LargeAreaHighGrayLevelEmphasis | 0.166 |
| 2 | original_glszm_LargeAreaLowGrayLevelEmphasis | 0.166 |
| 3 | original_glszm_LargeAreaEmphasis | 0.166 |
| 4 | wavelet-LLL_glszm_ZoneVariance | 0.125 |
| 5 | wavelet-HHH_glcm_ClusterProminence | 0.096 |
| 6 | wavelet-LLH_glcm_ClusterProminence | 0.094 |
| 7 | wavelet-HLL_glcm_ClusterProminence | 0.089 |
| 8 | log-sigma-2-0-mm-3D_glcm_ClusterProminence | 0.081 |
| 9 | wavelet-HHL_glcm_ClusterProminence | 0.078 |
| 10 | wavelet-HLH_glszm_ZoneVariance | -0.076 |
| 11 | wavelet-HLL_glcm_ClusterShade | 0.073 |
| 12 | wavelet-HLH_glcm_ClusterProminence | 0.070 |
| 13 | wavelet-HHH_glszm_ZoneVariance | -0.068 |
| 14 | log-sigma-5-0-mm-3D_glcm_ClusterProminence | 0.068 |
| 15 | wavelet-LLL_glcm_ClusterProminence | 0.067 |
| 16 | log-sigma-3-0-mm-3D_glszm_ZoneVariance | -0.067 |
| 17 | wavelet-HLH_glszm_LargeAreaEmphasis | -0.066 |
| 18 | wavelet-HHH_glszm_LargeAreaEmphasis | -0.066 |
| 19 | log-sigma-2-0-mm-3D_gldm_LargeDependenceLowGrayLevelEmphasis | 0.059 |
| 20 | wavelet-HLL_glszm_ZoneVariance | 0.057 |
| 21 | log-sigma-2-0-mm-3D_glszm_ZoneVariance | -0.055 |
| 22 | log-sigma-4-0-mm-3D_glcm_ClusterProminence | 0.055 |
| 23 | wavelet-LHH_glcm_ClusterProminence | 0.055 |
| 24 | wavelet-HLH_glszm_LargeAreaLowGrayLevelEmphasis | -0.054 |
| 25 | wavelet-LHL_glcm_ClusterProminence | 0.054 |
| 26 | wavelet-HHH_glcm_ClusterShade | 0.052 |
| 27 | log-sigma-3-0-mm-3D_glszm_LargeAreaEmphasis | -0.052 |
| 28 | log-sigma-2-0-mm-3D_glcm_ClusterShade | 0.052 |
| 29 | wavelet-HHL_glszm_LargeAreaHighGrayLevelEmphasis | 0.050 |
| 30 | log-sigma-3-0-mm-3D_glcm_ClusterProminence | 0.050 |

Table 1. Feat features and scores from the sixth principal component of *K*^trans^

Table 2. Feat features and scores from the fourth principal component of *K*^trans^

|  | Feat features | score |
| --- | --- | --- |
| 1 | original_glszm_LargeAreaHighGrayLevelEmphasis | 0.361 |
| 2 | original_glszm_LargeAreaLowGrayLevelEmphasis | 0.361 |
| 3 | original_glszm_LargeAreaEmphasis | 0.361 |
| 4 | wavelet-LLL_glcm_Imc2 | -0.258 |
| 5 | wavelet-HLL_glcm_Imc2 | -0.195 |
| 6 | wavelet-LHL_glcm_Imc2 | -0.182 |
| 7 | original_glszm_SmallAreaEmphasis | 0.145 |
| 8 | original_glszm_SmallAreaHighGrayLevelEmphasis | 0.145 |
| 9 | original_glszm_SmallAreaLowGrayLevelEmphasis | 0.145 |
| 10 | wavelet-LLH_glcm_Imc2 | -0.140 |
| 11 | wavelet-LLL_glszm_ZoneVariance | -0.082 |
| 12 | wavelet-HHL_glcm_Imc2 | -0.074 |
| 13 | wavelet-HLL_glszm_ZoneVariance | -0.070 |
| 14 | wavelet-LHL_gldm_GrayLevelNonUniformity | 0.067 |
| 15 | wavelet-LHL_glrlm_GrayLevelNonUniformity | 0.067 |
| 16 | wavelet-HHL_gldm_GrayLevelNonUniformity | 0.064 |
| 17 | wavelet-HLL_gldm_GrayLevelNonUniformity | 0.064 |
| 18 | wavelet-LLH_gldm_GrayLevelNonUniformity | 0.064 |
| 19 | wavelet-LLL_gldm_GrayLevelNonUniformity | 0.063 |
| 20 | wavelet-HLL_glrlm_GrayLevelNonUniformity | 0.063 |
| 21 | wavelet-LHH_gldm_GrayLevelNonUniformity | 0.063 |
| 22 | wavelet-LLL_glrlm_GrayLevelNonUniformity | 0.063 |
| 23 | wavelet-HHL_glrlm_GrayLevelNonUniformity | 0.062 |
| 24 | log-sigma-5-0-mm-3D_firstorder_Energy | 0.062 |
| 25 | log-sigma-5-0-mm-3D_firstorder_TotalEnergy | 0.062 |
| 26 | wavelet-HHH_gldm_GrayLevelNonUniformity | 0.062 |
| 27 | wavelet-HHH_glrlm_GrayLevelNonUniformity | 0.061 |
| 28 | wavelet-LLH_glrlm_GrayLevelNonUniformity | 0.061 |
| 29 | log-sigma-4-0-mm-3D_firstorder_Energy | 0.061 |
| 30 | log-sigma-4-0-mm-3D_firstorder_TotalEnergy | 0.061 |
| 31 | wavelet-HLH_gldm_GrayLevelNonUniformity | 0.061 |
| 32 | log-sigma-2-0-mm-3D_gldm_GrayLevelNonUniformity | 0.061 |
| 33 | wavelet-HLH_glrlm_GrayLevelNonUniformity | 0.060 |
| 34 | wavelet-LHH_glrlm_GrayLevelNonUniformity | 0.060 |
| 35 | log-sigma-2-0-mm-3D_glrlm_GrayLevelNonUniformity | 0.060 |
| 36 | log-sigma-3-0-mm-3D_firstorder_TotalEnergy | 0.060 |
| 37 | log-sigma-3-0-mm-3D_firstorder_Energy | 0.060 |
| 38 | log-sigma-2-0-mm-3D_firstorder_TotalEnergy | 0.060 |
| 39 | log-sigma-2-0-mm-3D_firstorder_Energy | 0.060 |
| 40 | wavelet-LHL_glszm_GrayLevelNonUniformity | 0.058 |
| 41 | log-sigma-3-0-mm-3D_gldm_GrayLevelNonUniformity | 0.058 |
| 41 | log-sigma-3-0-mm-3D_glrlm_GrayLevelNonUniformity | 0.057 |
| 42 | log-sigma-5-0-mm-3D_gldm_GrayLevelNonUniformity | 0.057 |
| 43 | wavelet-LLL_glszm_GrayLevelNonUniformity | 0.057 |
| 44 | log-sigma-4-0-mm-3D_gldm_GrayLevelNonUniformity | 0.057 |
| 45 | log-sigma-4-0-mm-3D_glrlm_GrayLevelNonUniformity | 0.056 |
| 46 | log-sigma-5-0-mm-3D_glrlm_GrayLevelNonUniformity | 0.056 |
| 47 | wavelet-HLL_glszm_GrayLevelNonUniformity | 0.056 |
| 48 | wavelet-HHL_glszm_GrayLevelNonUniformity | 0.055 |
| 49 | wavelet-LLH_glszm_GrayLevelNonUniformity | 0.053 |
| 50 | wavelet-HLH_glszm_GrayLevelNonUniformity | 0.053 |
| 51 | wavelet-LHH_glszm_GrayLevelNonUniformity | 0.052 |
| 52 | wavelet-HHH_glszm_ZoneVariance | 0.052 |
| 53 | log-sigma-2-0-mm-3D_glszm_GrayLevelNonUniformity | 0.051 |
| 54 | wavelet-HLH_glcm_Imc2 | -0.051 |
